# Trabecular Architecture Determines Impulse Propagation Through the Early Embryonic Mouse Heart

**DOI:** 10.3389/fphys.2018.01876

**Published:** 2019-01-08

**Authors:** Veronika Olejníčková, Barbora Šaňková, David Sedmera, Jiří Janáček

**Affiliations:** ^1^Department of Developmental Cardiology, Institute of Physiology of The Czech Academy of Sciences, Prague, Czechia; ^2^First Faculty of Medicine, Charles University, Prague, Czechia; ^3^Department of Biomathematics, Institute of Physiology of The Czech Academy of Sciences, Prague, Czechia

**Keywords:** cardiac conduction, mouse embryo, mathematical modeling, trabeculation, optical mapping

## Abstract

Most embryonic ventricular cardiomyocytes are quite uniform, in contrast to the adult heart, where the specialized ventricular conduction system is molecularly and functionally distinct from the working myocardium. We thus hypothesized that the preferential conduction pathway within the embryonic ventricle could be dictated by trabecular geometry. Mouse embryonic hearts of the Nkx2.5:eGFP strain between ED9.5 and ED14.5 were cleared and imaged whole mount by confocal microscopy, and reconstructed in 3D at 3.4 μm isotropic voxel size. The local orientation of the trabeculae, responsible for the anisotropic spreading of the signal, was characterized using spatially homogenized tensors (3 × 3 matrices) calculated from the trabecular skeleton. Activation maps were simulated assuming constant speed of spreading along the trabeculae. The results were compared with experimentally obtained epicardial activation maps generated by optical mapping with a voltage-sensitive dye. Simulated impulse propagation starting from the top of interventricular septum revealed the first epicardial breakthrough at the interventricular grove, similar to experimentally obtained activation maps. Likewise, ectopic activation from the left ventricular base perpendicular to dominant trabecular orientation resulted in isotropic and slower impulse spreading on the ventricular surface in both simulated and experimental conditions. We conclude that in the embryonic pre-septation heart, the geometry of the A-V connections and trabecular network is sufficient to explain impulse propagation and ventricular activation patterns.

## Introduction

In the adult heart of homeiotherm vertebrates (i.e., birds and mammals), the ventricular myocardium is activated by the His-Purkinje system, composed of the atrio-ventricular (A-V) bundle (of His), left and right bundle branches (of Tawara), and the network of Purkinje fibers (Sedmera and Gourdie, 2014). This network has a subendocardial component (in all species), and a transmural one in ungulates (Oosthoek et al., 1993; Ryu et al., 2009). In mice, as well as in humans, only the subendocardial fibers were described. The function of the His-Purkinje system is to assure a rapid and coordinated activation of the ventricular myocardium, resulting in its efficient contraction pushing the blood toward the outflow vessels, located at the base of the heart (Sedmera, 2011). The importance of this system is evidenced by ventricular dyssynchrony observed in patients with heart disease and left bundle branch block or right ventricular pacing (Ezzeddine and Dandamudi, 2018).

The conserved hallmark of the specialized, fast-conducting His-Purkinje network, is the specific expression of gap junction protein connexin40 (Cx40) in mammals, or its ortholog, connexin42, in birds (Gourdie et al., 1993). However, during the embryonic development, the expression of Cx40 is much broader (van Kempen et al., 1995; Van Kempen et al., 1996; Becker et al., 1998; Coppen et al., 2003), including all the trabeculae in both ventricles as well as the left ventricular compact layer at the pre-septation stages (Sedmera et al., 2003a). Consequently, the preferential conduction pathway of the electrical impulse traveling from the A-V junction should be determined at these stages mostly by tissue architecture, as the expression of gap junction channels is far more homogeneous and isotropic in immature cardiomyocytes (Angst et al., 1997). Thus, the arrangement of the trabecular network including its anisotropy, distance between junctions and prevailing orientation (Sedmera et al., 2000) together with its connections to the A-V canal seem to be critical in determining impulse spread through the embryonic pre-septated ventricle, as postulated already by de Jong et al. (1992) and experimentally verified by Reckova et al. (2003). The architectural differences between cardiac compartments are matched by molecular specification of the contractile protein phenotype (de Jong et al., 1987) and gap junction protein expression (Franco et al., 1998; Franco and Icardo, 2001). The transcriptional network responsible for differentiation of its individual components was subject of recent reviews (van Weerd and Christoffels, 2016; Park and Fishman, 2017).

The time of onset of the electrical activity in mouse heart was shown at ED8.5 (Chen et al., 2010). Advancement in the optical methods enabled the researchers to follow electrical propagation at the epicardial surface with sufficient spatial resolution (Sedmera et al., 2005; de la Rosa et al., 2013), distinguishing compartments with different conduction properties. To ensure effective pumping function with appropriate cardiac output, the slow impulse propagation in the A-V canal is followed by rapid activation of the embryonic ventricle (de Jong et al., 1992; Reckova et al., 2003). However, the epicardial maps reveal only surface manifestation of the ventricular conduction, lacking spatiotemporal information about activation of the internal architecture. Mathematical modeling of the adult heart linked conduction properties of the particular compartments with characteristics of 3-dimensional impulse propagation to create in-depth tissue activation view (Plank et al., 2009; Clayton et al., 2011; Lopez-Perez et al., 2015).

The adult left ventricle has been traditionally modeled as a body shaped between a sphere and a cylinder (Hutchins et al., 1978). Shape of the right ventricle is much more complex, and that of the embryonic one even more so. Early tubular heart could be well-approximated as a thick-walled cylinder (Taber, 2001), but for the trabeculated stages, it was shown that ignoring the trabeculae that form up to 80% of the myocardial mass and approximating the ventricle using a thick-shelled spherical model underestimates the wall stresses by several orders of magnitude (Buffinton et al., 2013). Thus, realistic trabecular geometry, obtainable using confocal microscopy or other 3D imaging techniques (Miller et al., 2005) should be used for modeling the embryonic heart, as the current computing power is well-able to handle such models.

The aim of this study was to characterize the conduction anisotropy resulting from the trabecular architecture in mouse embryonic hearts and compare the data of simulated physiological or ectopic impulse propagation with the actual epicardial activation maps from the same embryonic stages obtained from isolated hearts *in vitro* using optical mapping. Our simulated activation maps assuming constant speed of activation along the trabeculae, i.e., activation time proportional to geodetic distance from the impulse initiation position, showed activation patterns very similar to those experimentally obtained in the pre-septation hearts. These findings suggest that trabecular anisotropy together with the A-V myocardial connection is sufficient to explain impulse propagation and ventricular activation patterns in the embryonic heart.

## Materials and Methods

### Animals

All procedures performed on animals were in accordance with the ethical standards of the Charles University in Prague and were approved by the Animal Care and Use Committee of the First Faculty of Medicine.

The Nkx2.5:eGFP knock-in mice (Biben et al., 2000) were maintained in a heterozygous state since the homozygous one is lethal between embryonic days 9 and 10 (Lyons et al., 1995). The breeding pairs were caged overnight and the noon of the day when plug was discovered was considered embryonic day (ED) 0.5. Time-pregnant females were sacrificed by cervical dislocation and the embryos at desired stages (ED9.5–ED14.5) were rapidly dissected. The required heterozygous state was confirmed by eGFP expression in the heart, detected by epifluorescence microscope fitted with appropriate filter set (Olympus, Tokyo, Japan). The hearts of these embryos were purged clear of blood by orthograde perfusion through hepatic circulation with ice-cold cardioplegic solution and after that perfusion-fixed (Sedmera et al., 1997) in 4 % (wt/vol) in paraformaldehyde in phosphate buffer saline (PBS), followed by immersion in the same fixative for 48 h. After thorough rinsing with PBS, the dissected hearts were analyzed whole mount using tissue clearing methods optimized for cardiac tissues (Kolesova et al., 2016). Six embryos were sampled at each stage, three of which were processed for full 3D imaging and reconstruction.

### Optical Mapping

Optical mapping with voltage-sensitive dye di-4-ANEPPS was performed in wild type (C57/Bl6 strain) mouse embryos in the context of a previous study (Sankova et al., 2012) on hearts between ED9.5–18.5 (*N* = 14, 21, 10, 12, and 16 at ED9.5, 10.5, 11.5, 12.5, and 14.5, respectively). We have chosen to use the previously unpublished optical mapping data exclusively from the WT mice first to present the most physiological results without possible alterations due to transgene presence (and hence haploinsufficiency for the transcription factor Nkx2.5), and second, since it is essentially impossible to perform good quality perfusion on an isolated heart after optical mapping. As only summary data (statistics) for the WT animals were published, the figures presented here are all original. The most typical patterns prevailing at the respective stages were selected. Limited numbers of optically mapped WT and heterozygous embryos from the Nkx2.5:eGPF line (ED9.5 and ED12.5) as well as comparison with published data from the other labs (Rentschler et al., 2001) did not reveal any significant differences in frequency of dominant ventricular activation patterns; we thus consider them as typical for the species at particular developmental stages.

### Imaging Protocol

Trabeculae in the embryonic heart of Nkx2.5:eGFP mice were visualized in 3D using confocal microscope. CUBIC 2 protocol was applied for clearing the tissue to improve the imaging depth. Briefly, CUBIC 2 solution [N,N,N,N-tetrakis(2-hydroxypropyl)] ethylenediamine 25% (wt/vol), urea 25% (wt/vol), and polyethylene glycol mono-pisooctylphenyl ether (Triton X-100) 15% (wt/vol) in water was thoroughly mixed for 30 min at 37°C and then kept at room temperature. Dissected hearts were submerged and gently rocked in CUBIC 2 solution at 37°C for 2–3 days. We have previously showed that this clearing protocol results in the best embryonic heart tissue clearing while optimally preserving the eGFP signal at the same time (Kolesova et al., 2016).

Custom-made chambers (Miller et al., 2005) or cavity slides (Fisher Scientific, Pittsburgh, PA), filled with the CUBIC 2 solution and covered with a coverslip, were used for whole heart imaging. High-resolution images were obtained with a confocal system FluoView FV1000 fitted on an upright BX61 microscope (Olympus, Tokyo, Japan) with a 10x, 0.4 NA dry objective with lateral resolution of 0.4 pixels per micrometer and 2.5 μm z-step. The appropriate filter sets were used in order to properly capture eGFP (488 nm excitation) and autofluorescence (543 nm excitation) in sequential scanning mode.

### Image Processing

**Figure 1:**
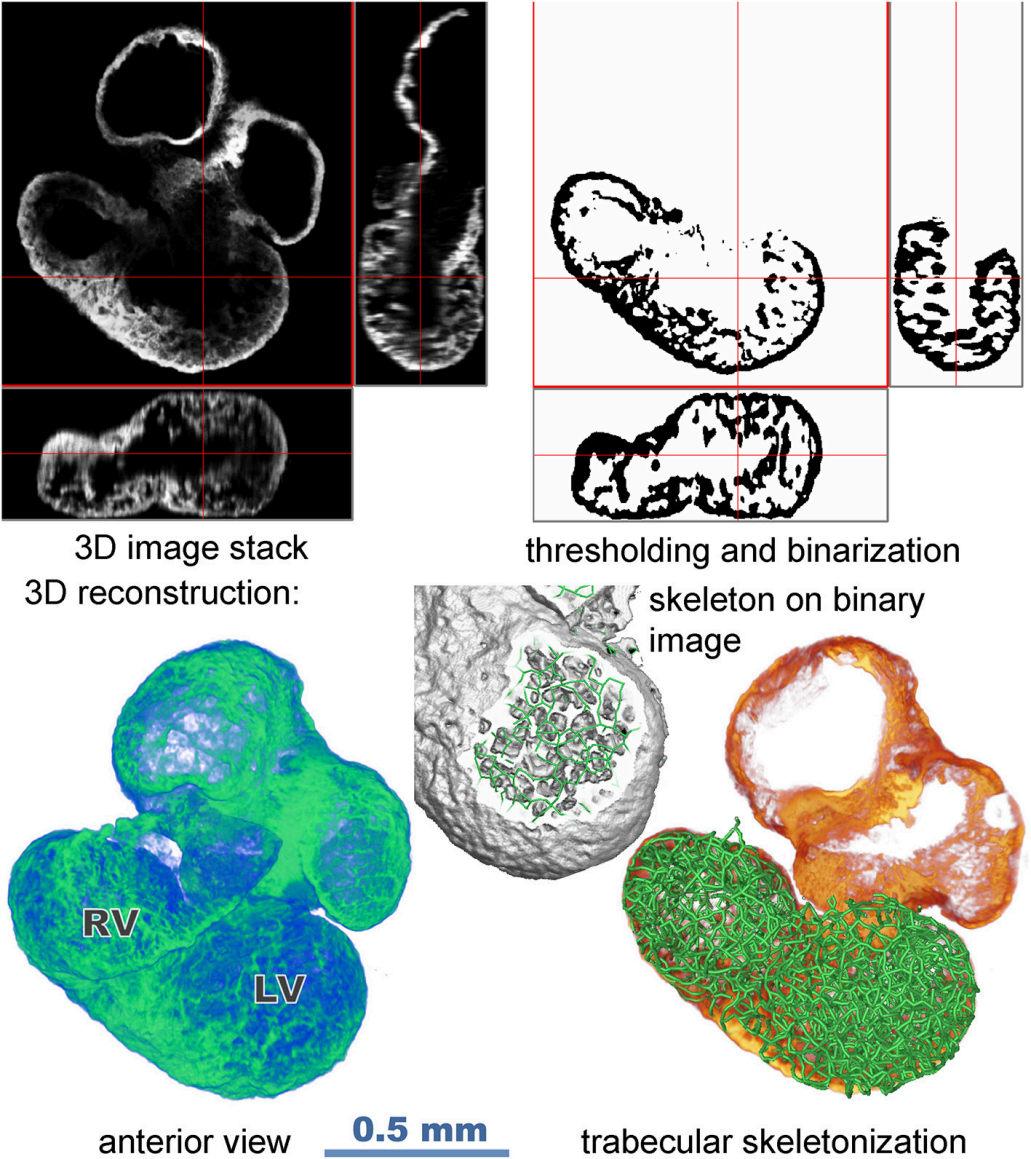
Flow chart illustrating data acquisition and image processing (stack merging, segmentation, reconstruction, and creation of skeleton model).

The field of view of the 10x, 0.4 NA dry objective necessitated 4 to 6 stacks in the x-y directions from both sides of the sample (Figure 1). Raw images containing 512 × 512 pixels in z stacks of 150–200 slices were imported into the Amira program (version 6.5, Visage Imaging GmbH, Berlin). Images from both sides of the sample were merged in the z and x–y directions to create a single stack (Figure 1). Correction was made for non-matching refractive indices of the objective (*n* = 1) and of the sample immersed in CUBIC 2 (*n* = 1.36) by resampling the voxels in x-y direction to obtain isotropic voxel size 3.4 μm.

The z-stacks of binary images of trabeculation were obtained by preprocessing the original image by the difference of 3D Gaussians filter (with parameters sigma set to 8 and 1.3 voxels) and thresholding. These were then skeletonized by 6-pass algorithm (Palagyi and Kuba, 1998). Branching points and endpoints in the binary stacks defined the nodes of the spatial graph while the digital curves in the binary stacks were converted to chains of line segments connecting the nodes. The septum, covered by trabecular network on both sides, was included in the model; however, the outer compact layer was excluded, as its skeletonization would results in artifacts. Since its thickness is rather small and locally uniform at the stages investigated (Sedmera et al., 2000), its exclusion is not likely to impede the resulting models. The small, disconnected components were removed from the graph. The resulting graph *G* = (*V, E*) consists of *I* vertices *V* connected by edges *E*

$$\begin{array}{l} V=\left\{v_{i}\in R^{3}|\;i=1\ldots I\right\}\\ E\subset \left\{\left(v_{i},v_{j}\right)|\;i,j=1\ldots I\right\} \end{array}$$

### Impulse Propagation Modeling

Assuming constant velocity of impulse propagation, the time of activation is proportional to geodesic distance from the site of activation. The geodesic distance from given set of the graph vertices was calculated using Dijkstra's algorithm (Dijkstra, 1959).

The distance value was interpolated and then visualized in Amira module Isosurface. The surface was ~0.1 mm below the surface of the heart. Morphological erosion was used for the surface definition.

### Local Anisotropy Analysis

We characterized the trabecular anisotropy on intermediate scale (tens of microns–bigger than the trabeculae and smaller than the ventricles) by the spatially homogenized tensor of directional conductivity (positive definite 3 × 3 matrix, averaged over neighborhood of the actual position, size of the neighborhood is tens of microns). Positive definite tensor (or matrix) is suitable for simplified description of the orientation distribution—important directions of the structure under study can be visualized by the ellipsoid with three axes. The main axis (length) is proportional to the square root of the eigenvalues of the tensor and oriented in orthogonal directions of the eigenvectors of the tensor. We used simulated conduction of heat as a tool for obtaining local anisotropy characteristics, not as a functional model of the impulse propagation. The idea behind is that both the directions of the individual trabeculae and their mutual positions and interconnections are important in the conduction activation as well as in the simulated heat transfer, so our method provides more relevant results than e.g., statistical averaging of the trabecular orientations.

Local anisotropy of the trabecular structure is characterized by directionality of heat conduction, or equivalently by directionality of Brownian motion. For this purpose we used the graph *G* = (*V, E*) with edges weighted by *r*-“resistance” (equal to reciprocal distance)

$$\begin{array}{l} r\left(v_{i},v_{j}\right)=∥v_{i}-v_{j}{∥}^{-1}. \end{array}$$

as the model of the trabecular structure.

The graph Laplacian Δ is *I* × *I* matrix with elements

$$\begin{array}{l} \Delta {\;}_{ii}=-\sum_{\left(v_{i},v_{j}\right)\in \;E}r\left(v_{i},v_{j}\right) \end{array}$$

Δ_*ij*_ = *r*(*v*_*i*_, *v*_*j*_) if *i* ≠ *j* for (*v*_*i*_, *v*_*j*_) ∈ *E*

and Δ_*ij*_ = 0 for (*v*_*i*_, *v*_*j*_) ∉ *E*

The Laplacian is employed in formulation of the heat equation on the graph:

$$\begin{array}{l} \Delta f\left(v,t\right)=\frac{\partial f}{\partial t}\left(v,t\right),v\in V,t\geq 0. \end{array}$$

λ_*n*_-eigenvalues and φ_*n*_-orthonormal eigenvectors of the graph Laplacian Δ are related by equations

$$\begin{array}{l} \Delta \varphi_{n}=\lambda_{n}\varphi_{n},n=1\ldots I \end{array}$$

so that $e^{\lambda_{n\;}t}\varphi_{n}\left(v\right)$ are generators of solutions of the heat equation. Heat kernel is:

$$\begin{array}{l} K\left(x,y,t\right)=\overset{I}{\underset{n=1}{\sum }}\varphi_{n}\left(x\right)\varphi_{n}\left(y\right)e^{\lambda_{n}t} \end{array}$$

The heat kernel second moments are:

$$\begin{array}{l} T_{lk}\left(y,t\right)=\underset{x\in V}{\sum }\overset{I}{\underset{n=1}{\sum }}\varphi_{n}\left(y\right)\varphi_{n}\left(x\right)e^{\lambda_{n}t}\;\;\;\left(x_{l}-y_{l}\right)\left(x_{k}-y_{k}\right),\\ l,k=1,2,3 \end{array}$$

Eigenvalues were calculated by DSBEVX routine in LAPACK using 64bit MS Visual C++ 10. Eigenvectors were calculated by inverse iterations:

$$\begin{array}{l} \left(\Delta -\lambda I\right)y=b_{m}\\ \;b_{m+1}=\pm \frac{y}{\left\|y\right\|} \end{array}$$

using LU decomposition from Numerical Recipes (Press et al., 1988), procedures bandec(), banbks(). Two thousands to three thousands eigenvalues for each graph were calculated.

**Figure 2:**
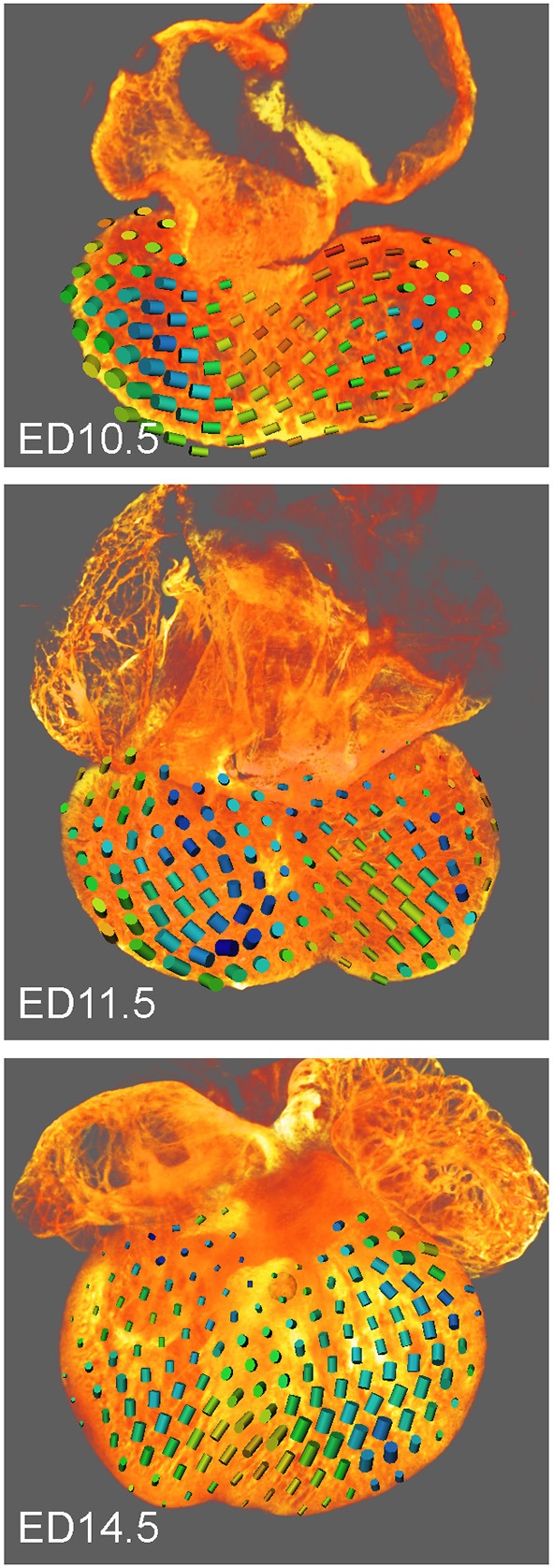
Tensor images showing trabecular orientation in mouse embryonic hearts at ED10.5, ED11.5, and ED14.5. The cylinder size is proportional to the conductivity, the shape and color (blue to red) reflect the trabecular anisotropy. Posterior views of fully reconstructed hearts; note differences between the septal area and the ventricles as well as increased isotropy at ED14.5.

The tensor of the heat kernel second moments *T*(*y, t*) was calculated for *t* = 30, the values were interpolated and visualized (Figure 2) in Amira 6.5 (FEI) using the module Tensor View.

The average of the interpolated tensors over the volume of the both ventricles was calculated and eigenvalues and eigenvectors of the average tensor were the calculated.

## Results

**Figure 3:**
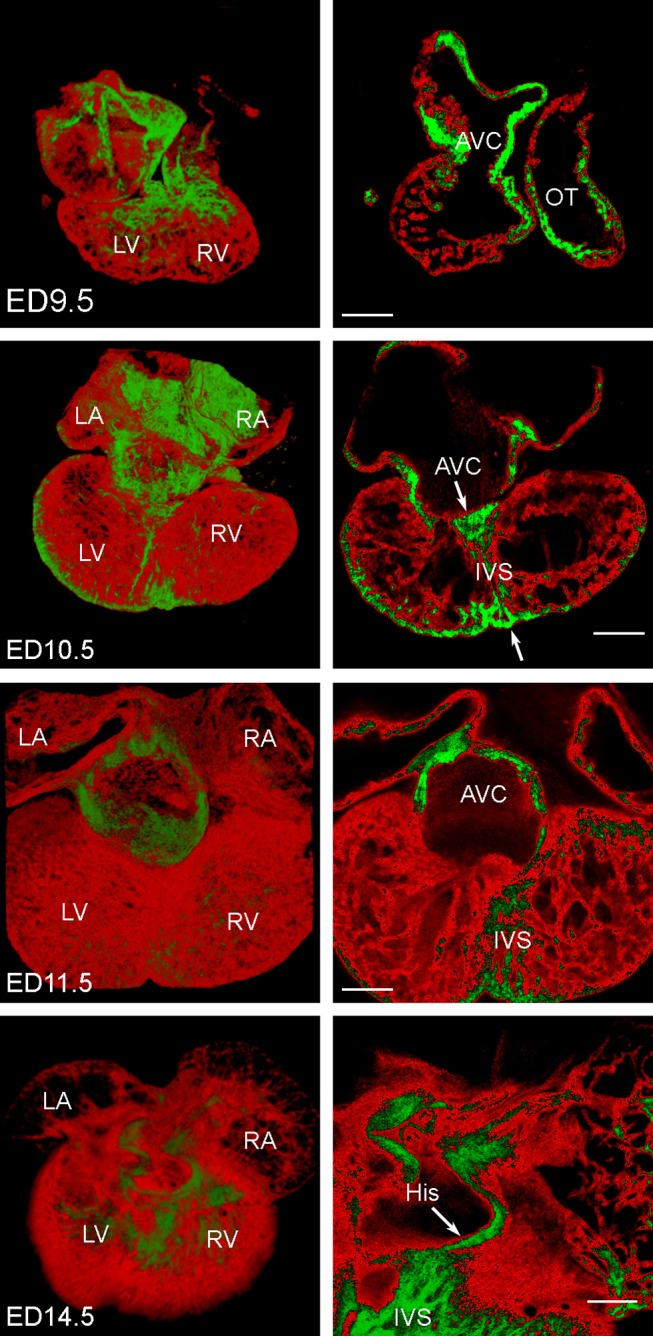
3D reconstructions of mouse embryonic Nkx2.5:eGFP hearts at ED9.5, ED10.5, ED11.5, and ED14.5. Expression of the eGFP in red, higher levels of expression in the atrioventricular canal (AVC), outflow tract (OT) and trabeculae in the right ventricle (RV) are highlighted in green; LA, left atrium; LV, left ventricle; RA, right atrium; scale bars 200 μm. All hearts are shown from the posterior aspect and 3D rendering is complemented by a single optical section through the A-V junction. His bundle is clearly present as a preferential dorsal atrioventricular conduction pathway terminating in the interventricular septum (IVS) at ED14.5, characterized by an increased expression of eGFP (and hence Nkx2.5). This pathway is, however, laid down much earlier (arrows and increased expression in the primary interventricular ring at ED10.5).

**Figure 4:**
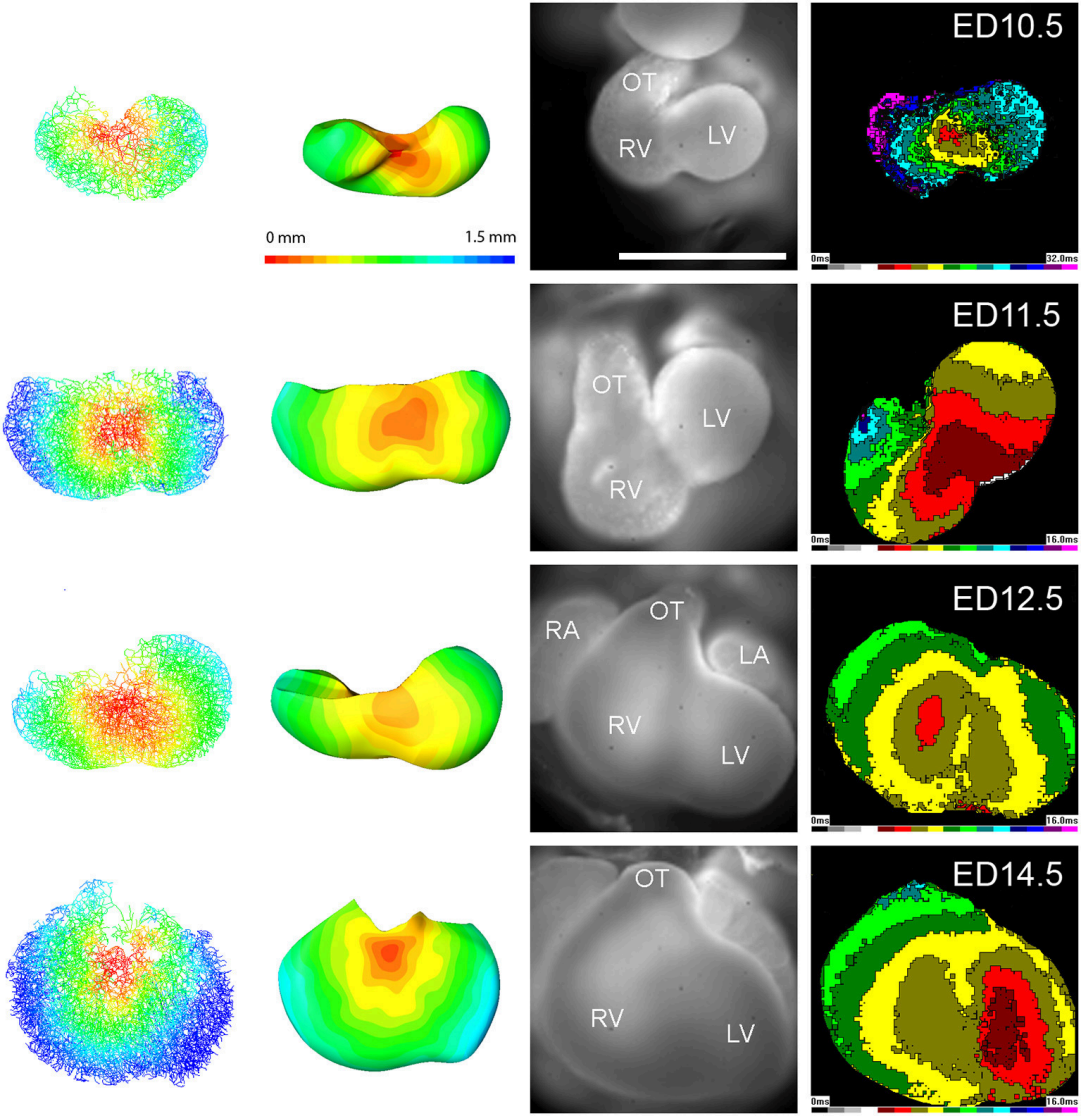
Examples of modeling of impulse propagation in mouse embryonic hearts at ED10.5, ED11.5, ED12.5, and ED14.5. Actual representative epicardial activation maps from wild type mouse embryonic hearts at the same stages are shown on the right side for comparison. The simulation and experimentally obtained map start to differ from ED12.5, suggesting a presence of an internal specialized conduction pathway. Scale bar 0.5 mm (all hearts were mapped at the same magnification).

Images of the Nkx2.5:eGFP mouse embryonic hearts between ED9.5 and ED14.5 were obtained and reconstructed (Figure 3). For stages past ED10.5, multiple 10x stacks (2–6) covering the heart were necessary, and for optimal imaging of deeper structures, imaging from the anterior and posterior aspect was combined. The A-V canal (as well as the outflow tract) showed stronger eGFP expression compared to rest of myocardium (Figure 3). High levels of Nkx2.5:eGFP fluorescence were noticed also in the left ventricular compact layer, and within the developing interventricular septum. This higher level of expression together with the A-V canal compact morphology allowed pinpointing its connection(s) to the septum and trabecular network (Figure 3). This junction, in the crest of the developing ventricular septum, was subsequently used for impulse initiation to model propagation of the activation wave (Figure 4).

**Figure 5:**
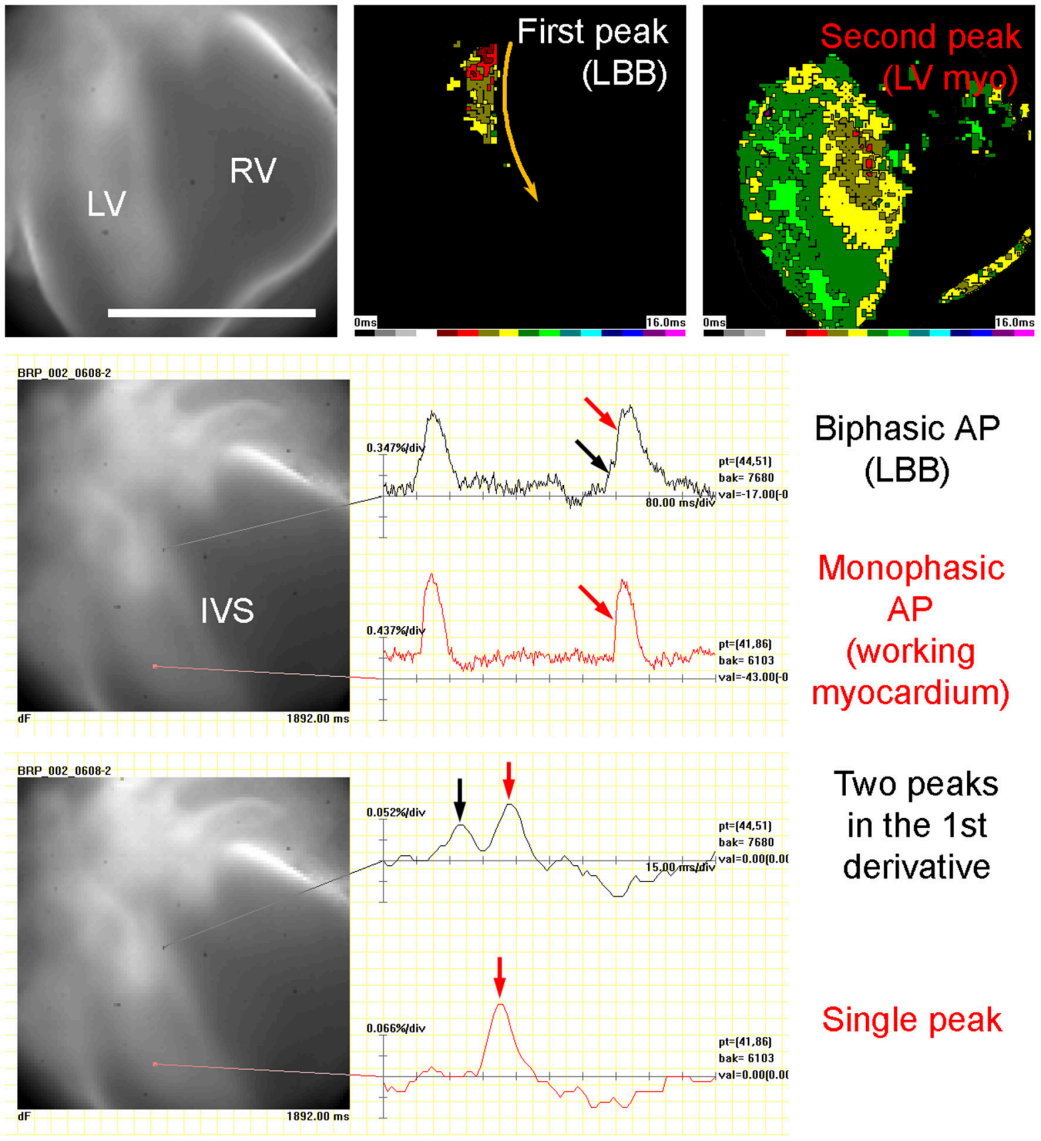
Experimental evidence of left bundle branch (LBB) functionality in ED14.5 mouse embryonic heart. Left side of the interventricular septum (IVS) shows a biphasic action potential (AP), which is made clearer after calculation of the first derivative that shows two peaks 24 ms apart. This allowed construction of two activation maps: the one from the first peak only shows the passing of the action potential through the LBB, while the second one, starting 30 ms milliseconds later, shows the activation of the entire left ventricular (LV) myocardium, which has a typical monophasic action potential and the first derivative with a single peak. Posterior view, scale bar 0.5 mm. Temporal scale is 80 ms per division (upper two traces) and 15 ms per division (lower two traces). For visualization, see the Supplemental Movie 1.

The 3D reconstruction also revealed that the A-V connection was progressively restricted from the whole A-V myocardium attaching to the left ventricle at ED9.5 to the exclusively posteriorly located communication with the interventricular septum at ED14.5 (Figures 1, 3, 5). Dominance of the posterior connection was evident from ED10.5 (Figure 3). Models of 3D impulse propagation through the trabecular network revealed in the epicardial surface a ring-like breakthrough pattern initiating in the developing ventricular septum up to ED11.5. The observations in the early stages were in good agreement with epicardial breakthrough patterns obtained by optical mapping. Both in the model as well as in the experimental maps, the activation was spreading from the forming interventricular septum–an area we termed the Primary Interventricular Ring in our previous work (Sankova et al., 2012). There was even an indication of two pathways along the sides, the one on the left being slightly faster than the right one in both model and experiment. However, at ED12.5, two epicardial breakthroughs started to appear in the optical maps, suggesting specialization of the interventricular conduction beyond the one indicated by the trabecular model. The epicardial activation at ED14.5 then differed considerably between the model and optical map. While the map revealed 2 centers at the ventricular apex in a vast majority (15 out of 16 hearts), the conduction model still showed isotropic activation spreading through the trabecular network from ventricular septum crest (Figure 4). This discrepancy was highlighted by revelation of preferential activation pathway along the interventricular septum through the developing left bundle branch (Figure 5 and Supplemental Movie 1) using optical mapping.

**Figure 6:**
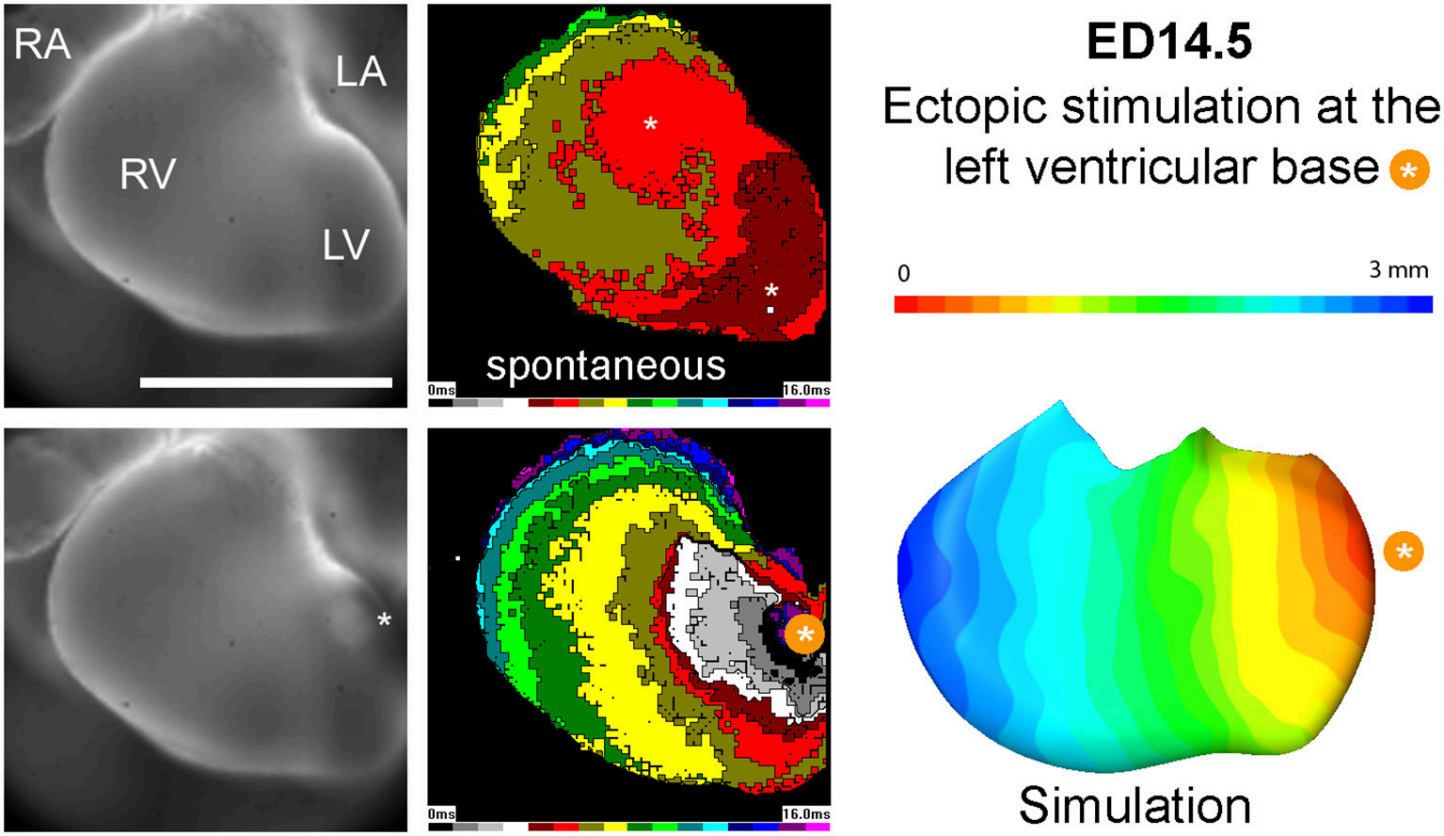
Propagation of spontaneous activation vs. paced beat at ED14.5. Epicardial breakthroughs in the sinus rhythm are indicated by white asterisks. Note increased time needed to activate the entire left ventricular surface for the paced beat. Simulation data (right) correlate well with the experimentally obtained activation map.

When in the model the impulse initiation point was placed on the compact myocardium of the basal part of the left ventricle, mimicking ectopic ventricular stimulation, simulated activation showed propagation in a concentric and isotropic manner. This conduction pattern was observed also in the optical maps (Figure 6).

The orientation of trabeculae, locally described by conductivity tensor (Figure 2), changed from horizontal at ED10.5 to vertical at ED14.5. The biggest eigenvalue of the average conductivity tensor was greater than the average of the other two eigenvalues by 11 percent at ED10.5, by 6 percent at ED11.5 and by 10 percent at ED14.5. It signifies that the degree of anisotropy was mild at all developmental stages.

## Discussion

We demonstrated that impulse propagation and ventricular activation patterns in the pre-septated heart could be explained by trabecular network and geometry of A-V connection. Simulation model following anisotropy of conduction resulting from 3D trabecular structure was able to predict epicardial activation patterns closely correlating with optical maps until the functional deployment of the ventricular conduction system (ED12.5). Exclusion of the compact layer in the skeleton model did not seem to influence the epicardial activation patterns (Figure 4), probably because of its small and uniform thickness at these stages (Sedmera et al., 2000) in contrast to the adult stages, where a complex fiber architecture is likely to influence transmural impulse propagation (Greiner et al., 2018). Reduction of the interventricular septum, which also develops a compact core from ED12.5 (Sankova et al., 2012) did not affect the modeled impulse propagation either, since the spontaneous activation is conducted through the trabeculae located on its surface (as validated experimentally, Figure 5), and even in the pacing mode, it did not behave like a significant current sink (Figure 6). Fractionated upstrokes were observed recently during the optical mapping of juvenile alligator heart in the interventricular septum, where they highlighted the dorsal preferential atrioventricular conduction pathway (Jensen et al., 2018). The ability to see such double upstrokes depends on good staining of both the internal and external myocardial layers, focal depth of the imaging system, and its spatial and temporal resolution. More detail from the depth of the heart could be revealed using line scan mode on a confocal microscope (Vostarek et al., 2014).

Since the expression of Cx40 until ED14.5 is homogeneous throughout the entire ventricular trabecular network (Sedmera et al., 2003a; Sankova et al., 2012), making this established CCS marker unsuitable for picking up preferential pathways (such as bundle branches and Purkinje fibers) at those stages, we used the Nkx2.5:eGFP mouse line to create isotropic 3D heart reconstruction. The Nkx2.5:eGFP staining within the AV canal and ventricle seems to be complementary to the Cx40:eGFP staining pattern. The strongest Cx40:eGFP expression was previously reported in the left ventricular trabeculae (Sedmera et al., 2003a; Sankova et al., 2012), where we found weaker Nkx2.5:eGFP fluorescence compared to the other cardiac compartments. In contrast, the strongest Nkx2.5:eGFP fluorescence was noticed in the AV canal, left ventricular compact layer, and within the developing interventricular septum (Figure 3), where the connexin40 expression is low or non-detectable (Sankova et al., 2012). This finding, supported by observed cellular hypoplasia of the peripheral conduction systems and reduced Cx40 expression in Nkx2.5 heterozygous mutants (Jay et al., 2004) is in a good agreement with the known impact of Nkx2.5 in differentiation of the conducting from myocardium working (Dupays et al., 2005; Harris et al., 2006; Mommersteeg et al., 2007). To explore the role of trabecular geometry in ventricular conduction, we studied stages from ED9.5, when cardiac tube starts to show ballooning of the atria and ventricles (Moorman et al., 2010), until the functional deployment of the bundle branches located on both sides of the ventricular septum at ED14.5 (Miquerol et al., 2004; Sankova et al., 2012).

Trabeculae appear on the luminal surface of the ventricles in a form of random open foam (Vuillemin and Pexieder, 1989; Sedmera and Thomas, 1996). “Random” means that the details of trabecular architecture in each individual are not completely genetically determined, i.e., have also a random component. Open foam is a technical term for a spatial structure without isolated voids. Closed foam contains cavities that are completely enveloped by the structure. This means, among other things, that all the intertrabecular spaces (Sedmera et al., 1998) are continuous with the ventricular lumen, as attested by the often-used description of the embryonic ventricle as “spongy” (Dusek et al., 1975; Taber et al., 1993; Jensen et al., 2012).

Parts of the trabeculae adjacent to the lumen gain certain degree of anisotropy during further maturation (Rychter and Rychterova, 1981; Sedmera et al., 1998), and their arrangement is species-dependent (Sedmera et al., 2000). The degree of the anisotropy was of interest for studies of the developing cardiac conduction system, because it could explain the known directionality of the excitation conduction. The degree of anisotropy in the present study seems to correspond with the gradual elongation of the ventricles (i.e., from sphere to ellipsoid), and the overall level of anisotropy is rather small, correlating well with our previous observations (Sedmera et al., 2000) as well as with another recent description (Captur et al., 2016) of mouse trabeculation as being rather isotropic (compared to other species). Assuming isotropic conduction with the only anisotropy due to trabecular geometry, similar to 2D *in vitro* data reported on cultured cardiomyocytes (Kucera et al., 1998), we have used the skeletonized model of the myocardium to calculate tensor and simulate impulse propagation through the ventricles from different activation points. If such an initiation site was placed at the crest of the interventricular septum, where the atrioventricular conduction pathway is known to be present at those stages (Reckova et al., 2003; Sedmera et al., 2003a), the epicardial breakthrough was observed in the apex as a ring-like breakthrough initiating in the interventricular location up to ED11.5. The conduction model showed the first site of epicardial breakthrough in ED12.5 heart at the apex, at the insertion of the septum. In contrast, the optical maps showed two breakthroughs, one on each side of the septum (Figure 4). The model thus, until to this developmental stage, matched the typical location observed in experimentally obtained activation maps. Then, however, the simulated internal conduction propagated up to the epicardial surface started to differ from the optically obtained activation breakthrough, and even more so at ED14.5. While the maps reflected functional connection of the forming bundle branches (Miquerol et al., 2004; Sankova et al., 2012) and showed two centers near the ventricular apex, the model revealed slow isotropic activation, suggesting thus major significance of the trabecular architecture in impulse propagation only in the pre-septated hearts.

Anisotropic conduction due to trabecular geometry could be further supported by recent observation from the spongy, but four chambered alligator hearts (Jensen et al., 2018). This group proposed the entire adult trabecular network as a morphological background for spreading of the electrical impulse through the ventricles, allowing the first epicardial breakthroughs to appear near the apex of the right and left ventricle in the morphological absence of specialized fast-conducting CCS. Further research addressed to exploring the transition of this conduction mode into specialized, partially or fully insulated, CCS in the adult mammalian heart is desirable.

In agreement with our previous data (Sankova et al., 2012; de la Rosa et al., 2013), we revealed the presence of A-V connections anteriorly and posteriorly in the early embryonic stages (ED10.5), but later on, only the posterior pathway persisted. The anterior connection, frequently present in the preseptation chick heart (Sedmera et al., 2004), was observed in the mouse only sporadically until ED11.5 (Sankova et al., 2012), correlating with gradual disappearance of this pathway (Figure 3). These findings correlated well with developing sequence in the activation maps. Interestingly, posteriorly located myocardial connection between the A-V canal and ventricular septum, favoring this side in electrical connection to the trabecular network, has been reported in crocodylian (Davies et al., 1952) as well as alligator heart (Jensen et al., 2018).

When impulse propagation was initiated on the compact myocardium of the basal part of the left ventricle, mimicking thus ectopic ventricular stimulation (de la Rosa et al., 2013), both the simulated activation as well as the experimentally obtained impulse propagation revealed concentric and isotropic manner of propagation (Figure 6). This is not surprising, since the orientation of the trabecular struts is perpendicular to the compact layer, with roughly equidistant spacing dictated by the diffusional needs for tissue oxygenation (Minot, 1901). Thus, myocardial geometry is able to explain ventricular activation sequence in both, spontaneous and paced rhythm, accounting for the impulse propagation in the absence of differentiated fast conduction pathway (i.e., before ED12.5). Such situation has been similarly described in poikilotherms (Sedmera et al., 2003b), where the connections between the A-V canal myocardium and trabecular sheets showed correlation with the first observed ventricular epicardial breakthrough during the spontaneous rhythm and much slower, isotropic impulse propagation was observed in the ectopic activation from the apical or basal ventricular part. More recent study in reptiles (Gregorovicova et al., 2018) likewise showed that myocardial geometry (i.e., orientation of the main trabecular sheets and their connection to the A-V canal) can explain distinct ventricular activation patterns in variously shaped hearts without any molecular specialization of such preferential pathways.

Trabeculae in the mouse embryonic ventricles form a very complex meshwork (Vuillemin and Pexieder, 1989), which is not easy to quantify. This problem was recently tackled using high-resolution episcopic microscopy (Captur et al., 2016). The authors used in the end fractal dimensions (essentially a measure of contour length) of the trabeculae obtained from 2D sections to quantify differences between the left and right ventricle, apico-basal or septo-lateral gradient, as well as differences in Mib1 mouse model of ventricular non-compaction. Essentially, the fractal number, corresponding to trabecular network complexity, correlated well with the visual observation of such gradients. Interestingly, in some stages and regions, significant quantitative differences were noted also between different mouse strains; however, presented figures do not show them as qualitatively striking and the authors themselves interpret them as variations of normally occurring developmental processes. While we used two different strains of mice in our study, we do not believe, also based upon histological examination of the hearts, that the potential differences are biologically significant and would dramatically alter the ventricular conduction parameters. Indeed, ventricular activation patterns were found to be remarkably conserved among the different lines we analyzed in the past 18 years, and any significant differences were linked to either readily observed anatomical disturbance (de la Rosa et al., 2013), or deletion of a functionally significant gene (Sankova et al., 2012).

In this study, we used simulated impulse propagation to uncover the internal impulse propagation through developing embryonic heart and correlated them to the ventricular surface activation. Simulation models of electrical activation of the adult mammalian hearts gained significant attention (Keener and Sneyd, 2009). Currently developed models used various levels of approximation to describe complex connections of the conduction system through highly resistant Purkinje-myocardial junctions to working myocardium and simulate electrophysiological behavior in 3 dimensions (Clayton et al., 2011; Bordas et al., 2012). Since the pre-septated embryonic myocardium exhibits low level of differentiation, the model of ventricular conduction should reflect impulse propagation more realistically. Here we show that the model is able to closely match epicardial activation maps obtained from optical mapping in spontaneous as well as paced rhythm until functional deployment of forming bundle branches. The model thus allows improving our understanding of electrophysiological behavior and internal ventricular conduction through the pre-septated embryonic heart during physiological development or propagation of externally applied electrical field.

The degree of trabecular anisotropy revealed by conductivity tensor is about 10 percent in all developmental stages under study. The trabecular anisotropy alone hence cannot explain the big differences in conduction speed between axial and transverse activation in ED14.5 that should be instead ascribed to occurrence of fast-conducting pathways. We thus conclude that the geometry of the A-V connections and trabecular network is sufficient to explain impulse propagation and ventricular activation patterns in the pre-septated heart.

## Study Limitations

The performance of meaningful statistics would require higher number of samples, and ideally also matching the maps and 3D rendering data in the same heart, which is impossible for technical reasons (different sample preparation and mouse background). Detailed quantitative analysis is thus unfortunately beyond the scope of this study.

The compact layer was not considered in the modeling. We suppose that the shortest path from the site of impulse initiation to the epicardium is through the trabeculae. Anatomically, at the stages analyzed, the compact layer is relatively thin without much regional variation (Sedmera et al., 2000) and shows, unlike at the postnatal stages, fairly isotropic arrangement until ED15.5.

The ability to record simultaneously optical signal from deep (septum, bundle branches, trabeculae) and superficial (epicardial) layers was limited by the spatial and temporal resolution of the system and ventricular wall transparency. More detailed assessment, although with much smaller spatial window, would be possible using confocal or light sheet microscopy.

## Author Contributions

DS conceived the study, interpreted the data, and wrote the manuscript. VO collected specimens, acquired confocal data, prepared figures, analyzed data, and edited the manuscript. BŠ performed optical mapping, generated optical maps, and edited the manuscript. JJ performed mathematical analysis, designed algorithms, performed calculations, generated models and images, and edited the manuscript.

### Conflict of Interest Statement

The authors declare that the research was conducted in the absence of any commercial or financial relationships that could be construed as a potential conflict of interest.

## Supplementary Material

Supplemental Movie 1 Visualization of the activation passing through the developing left bundle branch (first flash) before activating the remaining ventricular myocardium (second flash) in ED14.5 mouse heart, imaged from the posterior aspect.
